# Complete genome sequence of *Planctomyces limnophilus* type strain (Mü 290^T^)

**DOI:** 10.4056/sigs.1052813

**Published:** 2010-07-29

**Authors:** Kurt LaButti, Johannes Sikorski, Susanne Schneider, Matt Nolan, Susan Lucas, Tijana Glavina Del Rio, Hope Tice, Jan-Fang Cheng, Lynne Goodwin, Sam Pitluck, Konstantinos Liolios, Natalia Ivanova, Konstantinos Mavromatis, Natalia Mikhailova, Amrita Pati, Amy Chen, Krishna Palaniappan, Miriam Land, Loren Hauser, Yun-Juan Chang, Cynthia D. Jeffries, Brian J Tindall, Manfred Rohde, Markus Göker, Tanja Woyke, James Bristow, Jonathan A. Eisen, Victor Markowitz, Philip Hugenholtz, Nikos C. Kyrpides, Hans-Peter Klenk, Alla Lapidus

**Affiliations:** 1 DOE Joint Genome Institute, Walnut Creek, California, USA; 2 DSMZ - German Collection of Microorganisms and Cell Cultures GmbH, Braunschweig, Germany; 3 Los Alamos National Laboratory, Bioscience Division, Los Alamos, New Mexico, USA; 4 Biological Data Management and Technology Center, Lawrence Berkeley National Laboratory, Berkeley, California, USA; 5 Oak Ridge National Laboratory, Oak Ridge, Tennessee, USA; 6 HZI – Helmholtz Centre for Infection Research, Braunschweig, Germany; 7 University of California Davis Genome Center, Davis, California, USA

**Keywords:** stalk, multicellular rosettes, low salt tolerance, Gram negative, *Planctomycetales*, *Planctomycetes*, GEBA

## Abstract

*Planctomyces limnophilus* Hirsch and Müller 1986 belongs to the order *Planctomycetales*, which differs from other bacterial taxa by several distinctive features such as internal cell compartmentalization, multiplication by forming buds directly from the spherical, ovoid or pear-shaped mother cell and a cell wall which is stabilized by a proteinaceous layer rather than a peptidoglycan layer. Besides *Pirellula staleyi*, this is the second completed genome sequence of the family *Planctomycetaceae*. *P. limnophilus* is of interest because it differs from *Pirellula* by the presence of a stalk and its structure of fibril bundles, its cell shape and size, the formation of multicellular rosettes, low salt tolerance and red pigmented colonies. The 5,460,085 bp long genome with its 4,304 protein-coding and 66 RNA genes is a part of the *** G****enomic* *** E****ncyclopedia of* *** B****acteria and* *** A****rchaea * project.

## Introduction

Strain Mü 290^T^ (= DSM 3776 = ATCC 43296) is the type strain of *Planctomyces limnophilus* [1]. Currently, there are six species placed in the genus *Planctomyces* [2], the type species of which is *P. bekefii* [3-5]. The type species was initially described as a fungus under the International Code of Botanical Nomenclature [3,6]. The species *P. guttaeformis* and *P. stranskae* were also initially described as fungi, with their names being revived under the Bacteriological Code in 1984 [7]. The genus name derives from the Greek words ‘planktos’, wandering, floating, and ‘mukês’ meaning ‘fungus’ to indicate a floating fungus [3], reflecting their initial descriptions as members of the fungi. The species epithet derives from the Greek words ‘limnos’, lake, and ‘philos’, friend, loving, to indicate lake-loving [1]. Strain Mü 290^T^ together with another strain (strain 279 = DSM 1115) have been isolated from the freshwater lake Plußsee in Holstein, Germany [1]. Other strains of *P. limnophilus* have been isolated from Schrevenpark, Lake Mondsee, a ‘cattle manure’ (all near Kiel, Germany), and leakage water from a (industrial) compost heap (probably also in Germany) and were originally stored at the IFAM collection (Institut für Allgemeine Mikrobiologie, University of Kiel, Germany) [8].

The *rpoN* gene from *P. limnophilus* has been used in complementation studies in order to demonstrate the range of phylogenetic groups within the domain *Bacteria* that are known to contain the alternative sigma factor σ^54^ [9]. *P. limnophilus* strain Mü 290^T^ has also been utilized to demonstrate the widespread presence of the *dnaK* (HSP70) multigene family in members of the orders *Planctomycetales* and *Verrucomicrobiales* [10]. Quite early, in 1996, a physical map of the genome of strain Mü 290^T^ had been obtained [11]. *P. limnophilus* strain Mü 290^T^ was also utilized in a comparative analysis of ribonuclease P RNA of the *Planctomycetes* [12]. Here we present a summary classification and a set of features for *P. limnophilus* Mü 290^T^, together with the description of the complete genomic sequencing and annotation.

## Classification and features

This organism has a distinct cell cycle, with sessile mother cells forming stalks that attach to surfaces or to other stalks and motile daughter cells that bud from the mother cell. Mother cells are spherical to ovoid with stalks composed of twisted fibrils [1]. The diameter of the mother cell is 1.1 to 1.5 µm. Multiplication occurs by budding on the distal cell pole, yielding daughter cells which are monotrichously and polarly flagellated [1]. The carbon sources D-glucose, D-galactose, maltose, cellobiose, N-acetyl glucosamine are utilized (0.1% w/v) (Table 1), but not glucuronic acid, D-fructose, D-ribose, mannitol, starch, dextrin, inulin, salicin, pyruvate, citrate, α-oxoglutarate, succinate, fumarate, malate, formamide, methylamine·HCl (0.136%), formate (0.136%), urea (0.09%), methane (0.5%), methanol (0.4%), ethanol (0.4%), lactate, acetate, propionate, tartrate, glutarate, caproate, phtalate, glycerol (0.186%), L-arginine, L-aspartate, DL-alanine, L-glutamate, L-glycine, L-histidine, L-leucine, DL-phenylalanine, L-proline, and L-serine [1]. There is no aerobic acid formation from D-glucose, saccharose, D-fructose, maltose, D-galactose and mannitol, nor is there anaerobic acid formation from D-fructose or mannitol. However, there is anaerobic acid formation from D-glucose, saccharose, maltose or galactose [1]. Anaerobic gas formation on Hugh-Leifson medium was not reported. (NH_4_)_2_SO_4_ was utilized as a nitrogen source , but not NaNO_2_ (0.2 - 0.7%), NaNO_3_ (0.2 - 0.85%), methylamine·HCl (0.675%) or urea (0.46%) [1]. Strain Mü 290^T^ does not require vitamin supplements. It is reported to perform dissimilatory nitrate reduction, gelatin liquefaction, H_2_S formation and is tolerant to 30 vol% CO [1]. However, strain Mü 290^T^ is negative for decarboxylation of lysine or arginine, deamination of phenylalanine or lysine, oligocarbophilic growth, urease, nitrification, assimilatory nitrate reduction, anaerobic gas formation with nitrate, formation of acetoin (up to 27 d) or indole, growth in or changes of litmus milk, tolerance of 50 vol% CO, and extracellular DNase [1].

**Table 1:** Classification and general features of *P. limnnphilus* Mü 290^T^ according to the MIGS recommendations [13]

**MIGS ID**	**Property**	**Term**	**Evidence code**
	Current classification	Domain *Bacteria*	TAS [14]
		Phylum *Planctomycetes*	TAS [15]
		Class *Planctomycetacia*	TAS [15]
		Order *Planctomycetales*	TAS [16]
		Family *Planctomycetaceae*	TAS [16]
		Genus *Planctomyces*	TAS [3-5,17]
		Species *Planctomyces limnophilus*	TAS [1]
		Type strain Mü 290	TAS [1]
	Gram stain	negative	TAS [1]
	Cell shape	spherical to ovoid mother cells with stalks composed of twisted fibrils, sessile mother cells produces motile daughter cells	TAS [1]
	Motility	monotrichously and polarly flagellated	TAS [1]
	Sporulation	non-sporulating	TAS [1]
	Temperature range	17°C–39°C	TAS [1]
	Optimum temperature	30-32°C	TAS [1]
	Salinity	< 1% NaCl	TAS [1]
MIGS-22	Oxygen requirement	aerobic	TAS [1]
	Carbon source	D-glucose, D-galactose, maltose, cellobiose, N-acetyl glucosamine	TAS [1]
	Energy source	carbohydrates	TAS [1]
MIGS-6	Habitat	lakes and pools	TAS [1]
MIGS-15	Biotic relationship	free-living	TAS [1]
MIGS-14	Pathogenicity	not reported	NAS
	Biosafety level	1	TAS [18]
	Isolation	surface water of a lake	TAS [1]
MIGS-4	Geographic location	Lake Plußsee, Holstein, Germany	TAS [1,19]
MIGS-5	Sample collection time	1977 or before	TAS [1,19]
MIGS-4.1 MIGS-4.2	Latitude Longitude	54.182 10.445	NAS
MIGS-4.3	Depth	surface waters	NAS
MIGS-4.4	Altitude	about sea level	NAS

Evidence codes - IDA: Inferred from Direct Assay (first time in publication); TAS: Traceable Author Statement (i.e., a direct report exists in the literature); NAS: Non-traceable Author Statement (i.e., not directly observed for the living, isolated sample, but based on a generally accepted property for the species, or anecdotal evidence). These evidence codes are from of the Gene Ontology project [20]. If the evidence code is IDA, then the property was directly observed by one of the authors or an expert mentioned in the acknowledgements

**Figure 1:**
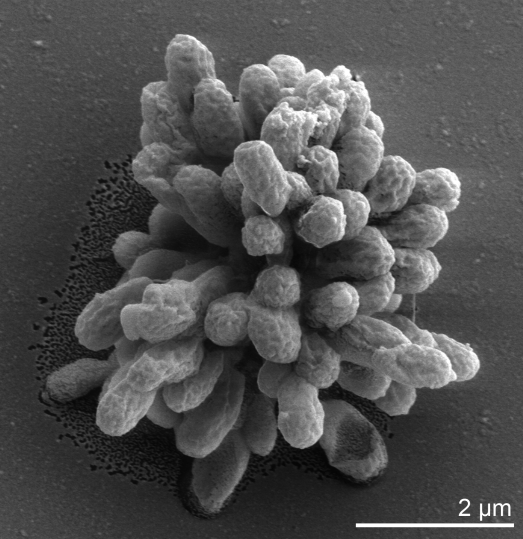
Scanning electron micrograph of *P. limnophilus* Mü 290^T^

As a member of the order *Planctomycetales*, *P. limnophilus* strain Mü 290^T^ is characterized by several distinctive morphological features such as rigid stalk fibers and the formation of multicellular rosettes (Figure 1) [1]. Further studies on another *Planctomyces* species, *P. maris* [21], revealed internal cell compartmentalization into the nucleoid, paryphoplasm, and a large ovoid central region [22]. The 16S rRNA gene sequence similarity values among isolates of the currently described species of this genus are sufficiently divergent to consider a re-examination of their taxonomy, e.g. the sequences of the two other type strains in the genus, *P. maris* [21] and *P. brasiliensis* [23] each share only 84.9% sequence identity with strain Mü 290^T^ [the other three species in this genus are currently without an available type strain], whereas the other type strains from the family *Planctomycetaceae* share 78.8 to 82.8% sequence identity with strain Mü 290^T^ [24]. This view is indirectly supported by the establishment of the genus *Schlesneria*, which is placed within the radiation of the genus *Planctomyces* [25] with up to 88.2% sequence identity with strain Mü 290^T^. Any taxonomic re-arrangements are linked to the absence of suitable biochemical/physiological, gene sequence and chemotaxonomic data for the type species of the genus and two additional species. Uncultured clone sequences similar to the 16S rRNA gene sequence from *P. limnophilus* were obtained from earthworm gut (98%, FJ542967) [26], however, metagenomic surveys do not surpass 83% sequence similarity (status June 2010).

**Figure 2:**
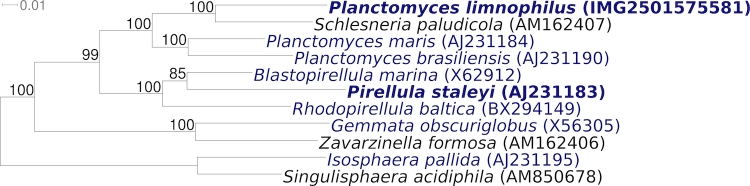
Phylogenetic tree highlighting the position of *P. limnophilus* Mü 290^T^ relative to the type strains of the other species within the genus and to the type strains of the other genera within the family *Planctomycetaceae*. The tree was inferred from 1,336 aligned characters [27,28] of the 16S rRNA gene sequence under the maximum likelihood criterion [29] and rooted in accordance with the current taxonomy [30]. The branches are scaled in terms of the expected number of substitutions per site. Numbers above branches are support values from 1,000 bootstrap replicates [31] if larger than 60%. Lineages with type strain genome sequencing projects registered in GOLD [32] are shown in blue, published genomes in bold, e.g. the recently published GEBA genome of *Pirellula staleyi* [33]. 16S rRNA gene sequences are not available for strains of the species *P. bekefii, P. guttaeformis* or *P. stranskae*, all of which are typified by descriptions and were initially described as fungi [1,7]. The name *P. gracilis* was also initially described as a fungus, but the name has not been validly published under the Bacteriological Code. Starr *et al.* [34] considered this organism not be to a planctomycete.

Figure 2 shows the phylogenetic neighborhood of *P. limnophilus* Mü 290^T^ in a 16S rRNA based tree. The sequences of the two identical 16S rRNA gene copies differ by one nucleotide from the previously published 16S rRNA sequence (X62911) generated from IFAM 1008, which contains one ambiguous base call.

### Chemotaxonomy

The genus *Planctomyces* lacks muramic acid and diaminopimelic acid, as was determined for *P. maris* [35]. However, a large amount of aspartic acid was found in whole cell hydrolysates [35]. Instead of containing peptidoglycan, the 10% SDS resistant cell envelope consisted almost entirely of protein which is rich in proline and cysteine and is stabilized to a high degree by disulfide bonds [36]. Comparable data are not available for *P. limnophilus*. The fatty acids in the polar lipids of strain Mü 290^T^ are C_16:0_ (46.6%), C_18:1ω9c_ (20.6%),C_16:1ω7c_ (18.4%), C _18:1ω7c_ (5.5%), C _15:0_ (1.0%), C _17:0_ (1.7%), C _18:0_ (1.0%), C _17:1ω8c_ (2.6), and C _20:1ω9c_ (1.3%) [37]. A similar fatty acid composition was reported by Kulichevskaya *et al*. [25], who also reported the presence of long chain, saturated alcohols and diols. The dominant lipopolysaccharide hydroxy fatty acid of strain Mü 290^T^ are C_3-OH 14:0_ (74.1%),C_3-OH 20:0_ (22.5%), and C_3-OH 18:0_ (3.4%) [37]. The sole respiratory lipoquinone is MK-6, a feature of all members of the aerobic members of the family *Planctomycetaceae* examined to date [38]. Like all members of Sittig and Schlesner’s group 3 *Planctomycetes* the type strain produced phosphatidylmonomethylethanolamine, phosphatidyldimethyl-ethanolamine, a glycolipid and smaller amounts of phosphatidylglycerol, phosphatidylcholine and bisphosphatidylglycerol [38].

A survey on the cellular polyamine pattern of members of the order *Planctomycetales* revealed *P. limnophilus* strain Mü 290^T^ to contain a large amount of putrescine and a relatively small amount of spermidine [8].

## Genome sequencing and annotation

### Genome project history

This organism was selected for sequencing on the basis of its phylogenetic position [39], and is part of the *** G****enomic* *** E****ncyclopedia of* *** B****acteria and* *** A****rchaea * project [40]. The genome project is deposited in the Genome OnLine Database [32] and the complete genome sequence is deposited in GenBank. Sequencing, finishing and annotation were performed by the DOE Joint Genome Institute (JGI). A summary of the project information is shown in Table 2.

**Table 2:** Genome sequencing project information

**MIGS ID**	**Property**	**Term**
MIGS-31	Finishing quality	Finished
MIGS-28	Libraries used	Two genomic libraries: one Sanger 8 kb pMCL200 library, one 454 pyrosequence standard library
MIGS-29	Sequencing platforms	ABI3730, 454 GS FLX, Illumina GAii
MIGS-31.2	Sequencing coverage	4.8× Sanger; 19.1× pyrosequence
MIGS-30	Assemblers	Newbler version 1.1.02.15, PGA
MIGS-32	Gene calling method	Prodigal 1.4, GenePRIMP
	INSDC ID	CP001744 chromosome CP001745 plasmid
	Genbank Date of Release	May 17, 2010
	GOLD ID	Gc01328
	NCBI project ID	29411
	Database: IMG-GEBA	2501533208
MIGS-13	Source material identifier	DSM 3776
	Project relevance	Tree of Life, GEBA

### Growth conditions and DNA isolation

*P. limnophilus* Mü 290^T^, DSM 3776, was grown in DSMZ medium 621 (PYGV medium) [41] at 28°C. DNA was isolated from 0.5-1 g of cell paste using Qiagen Genomic 500 DNA Kit (Qiagen, Hilden, Germany) following the standard protocol as recommended by the manufacturer, with doubled incubation time (1 hour) for cell lysis.

### Genome sequencing and assembly

The genome was sequenced using a combination of Sanger and 454 sequencing platforms. All general aspects of library construction and sequencing can be found at the JGI website (http://www.jgi.doe.gov/). Pyrosequencing reads were assembled using the Newbler assembler version 1.1.02.15 (Roche). Large Newbler contigs were broken into 6,078 overlapping fragments of 1,000 bp and entered into assembly as pseudo-reads. The sequences were assigned quality scores based on Newbler consensus q-scores with modifications to account for overlap redundancy and adjust inflated q-scores. A hybrid 454/Sanger assembly was made using the parallel phrap assembler (High Performance Software, LLC). Possible mis-assemblies were corrected and gaps between contigs were closed by editing in Consed, by custom primer walks from sub-clones or PCR products. A total of 18 Sanger finishing reads were produced to close gaps, to resolve repetitive regions, and to raise the quality of the finished sequence. Illumina reads were used to improve the final consensus quality using an in-house developed tool (the Polisher) [42]. The error rate of the completed genome sequence is less than 1 in 100,000. Together, the combination of the Sanger and 454 sequencing platforms provided 23.9× coverage of the genome. The final assembly contains 43,393 Sanger reads and 544,012 pyrosequencing reads.

### Genome annotation

Genes were identified using Prodigal [43] as part of the Oak Ridge National Laboratory genome annotation pipeline, followed by a round of manual curation using the JGI GenePRIMP pipeline [44]. The predicted CDSs were translated and used to search the National Center for Biotechnology Information (NCBI) nonredundant database, UniProt, TIGRFam, Pfam, PRIAM, KEGG, COG, and InterPro databases. Additional gene prediction analysis and functional annotation was performed within the Integrated Microbial Genomes - Expert Review (IMG-ER) platform [45].

## Genome properties

**Figure 3:**
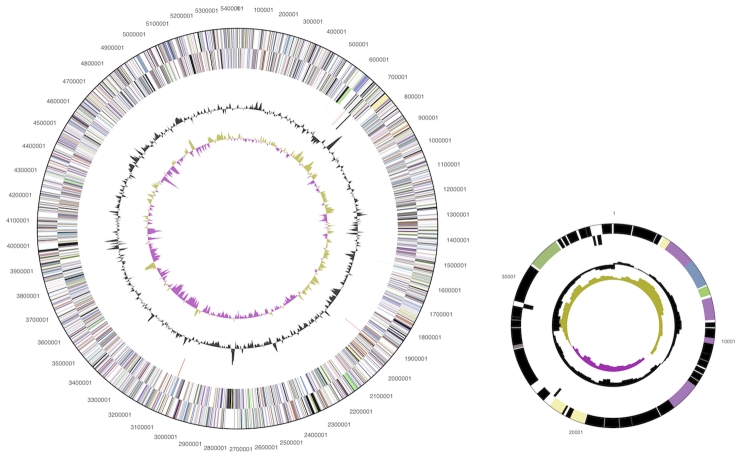
Graphical circular map of the chromosome and the plasmid (not drawn to scale). From outside to the center: Genes on forward strand (color by COG categories), Genes on reverse strand (color by COG categories), RNA genes (tRNAs green, rRNAs red, other RNAs black), GC content, GC skew.

The genome consists of a 5,460,075 bp long chromosome and a 37,010 bp long plasmid with a total G+C content of 53.7% (Table 3 and Figure 3). Of the 4,370 genes predicted, 4,304 were protein-coding genes, and 66 RNAs; 46 pseudogenes were also identified. The majority of the protein-coding genes (53.9%) were assigned a putative function while the remaining ones were annotated as hypothetical proteins. The distribution of genes into COGs functional categories is presented in Table 4.

**Table 3:** Genome Statistics

**Attribute**	**Value**	**% of Total**
Genome size (bp)	5,446,085	100.00%
DNA coding region (bp)	4,619,194	84.60%
DNA G+C content (bp)	2,931,217	53.68%
Number of replicons	2	
Extrachromosomal elements	1	
Total genes	4,370	100.00%
RNA genes	66	1.51%
rRNA operons	1	
Protein-coding genes	4,304	98.49%
Pseudo genes	46	1.05%
Genes with function prediction	2,355	53.89%
Genes in paralog clusters	353	8.08%
Genes assigned to COGs	2,463	56.36%
Genes assigned Pfam domains	2,691	61.58%
Genes with signal peptides	1,008	23.07%
Genes with transmembrane helices	1,126	25.77%
CRISPR repeats	1	

**Table 4:** Number of genes associated with the general COG functional categories

**Code**	**value**	**%age**	**Description**
J	149	5.2	Translation, ribosomal structure and biogenesis
A	0	0.0	RNA processing and modification
K	172	6.0	Transcription
L	141	4.9	Replication, recombination and repair
B	1	0.0	Chromatin structure and dynamics
D	22	0.8	Cell cycle control, cell division, chromosome partitioning
Y	0	0.0	Nuclear structure
V	67	2.3	Defense mechanisms
T	168	5.8	Signal transduction mechanisms
M	166	5.8	Cell wall/membrane/envelope biogenesis
N	150	5.2	Cell motility
Z	0	0.0	Cytoskeleton
W	0	0.0	Extracellular structures
U	178	6.2	Intracellular trafficking, secretion, and vesicular transport
O	124	4.3	Posttranslational modification, protein turnover, chaperones
C	161	5.6	Energy production and conversion
G	154	5.4	Carbohydrate transport and metabolism
E	192	6.7	Amino acid transport and metabolism
F	54	1.9	Nucleotide transport and metabolism
H	127	4.4	Coenzyme transport and metabolism
I	73	2.5	Lipid transport and metabolism
P	148	5.1	Inorganic ion transport and metabolism
Q	54	1.9	Secondary metabolites biosynthesis, transport and catabolism
R	370	12.9	General function prediction only
S	206	7.2	Function unknown
-	1,907	43.6	Not in COGs
